# The Many Faces of Protein–Protein Interactions: A Compendium of Interface Geometry

**DOI:** 10.1371/journal.pcbi.0020124

**Published:** 2006-09-29

**Authors:** Wan Kyu Kim, Andreas Henschel, Christof Winter, Michael Schroeder

**Affiliations:** 1 Bioinformatics Group, Biotechnological Centre, Technische Universität Dresden, Dresden, Germany; 2 European Bioinformatics Institute, Wellcome Trust Genome Campus, Cambridge, United Kingdom; University of California San Diego, United States of America

## Abstract

A systematic classification of protein–protein interfaces is a valuable resource for understanding the principles of molecular recognition and for modelling protein complexes. Here, we present a classification of domain interfaces according to their geometry. Our new algorithm uses a hybrid approach of both sequential and structural features. The accuracy is evaluated on a hand-curated dataset of 416 interfaces. Our hybrid procedure achieves 83% precision and 95% recall, which improves the earlier sequence-based method by 5% on both terms. We classify virtually all domain interfaces of known structure, which results in nearly 6,000 distinct types of interfaces. In 40% of the cases, the interacting domain families associate in multiple orientations, suggesting that all the possible binding orientations need to be explored for modelling multidomain proteins and protein complexes. In general, hub proteins are shown to use distinct surface regions (multiple faces) for interactions with different partners. Our classification provides a convenient framework to query genuine gene fusion, which conserves binding orientation in both fused and separate forms. The result suggests that the binding orientations are not conserved in at least one-third of the gene fusion cases detected by a conventional sequence similarity search. We show that any evolutionary analysis on interfaces can be skewed by multiple binding orientations and multiple interaction partners. The taxonomic distribution of interface types suggests that ancient interfaces common to the three major kingdoms of life are enriched by symmetric homodimers. The classification results are online at http://www.scoppi.org.

## Synopsis

The behaviour of biological systems is governed by protein interactions. Considerable effort has already been dedicated to characterise individual proteins and their evolution. As a next step, researchers need to understand the characteristics, dynamics, and evolution of complex networks of proteins. While many experimental techniques determine high-throughput protein–protein interactions, only few provide structural insights into the actual interfaces. The authors provide a comprehensive compendium and classification of these structural interfaces. To this end, they design a fast and accurate algorithm, which they apply to all known structural interactions. As a result, they shed light on the geometry and the evolution of protein interfaces. Their analysis reveals that 40% of protein interactions between homologues associate in multiple orientations. This has, in particular, implications for gene fusion events detected by conventional sequence homology: for one-third of these genes, the fused and nonfused proteins associate in alternative binding orientations. The classification also shows that any evolutionary analysis, such as interface conservation, can be skewed by multiple binding orientations and interaction partners. Hub proteins, which are highly connected to many other proteins in interaction networks, are shown to use distinct surfaces, or faces, for different partners. Interestingly, some proteins develop many different faces for the same partner (e.g., long-chain cytokines and fibronectin), and others use the same face for evolutionary unrelated partners (e.g., the PUA domain family). Finally, the authors show that ancient interfaces, which appear in all three kingdoms of life, are dominated by symmetric homodimers, reflecting the direction of evolution from symmetric to asymmetric or heteromeric.

## Introduction

Protein tertiary and quaternary structures often provide a deep insight into a protein's function and its underlying mechanism. Though the number of available structures is growing rapidly, including multidomain proteins and protein complexes, solving large protein structures is still challenging. If the structures of the component domains and subunits are known, systematic docking [1] or multimeric threading [2] may be tried, but both approaches require enormous computation for a genomewide application. Aloy and colleagues proposed that close homologues tend to interact in similar orientations [3]. This observation provided a theoretical basis for a breakthrough in modelling protein 3-D complexes by combining interactions from known structures [4]. However, some proteins associate in multiple orientations even between close homologs, as shown in lectins [5] and bacterial chemotaxis-related proteins [6]. The multiplicity of binding orientation is, in fact, shown to be widespread among different domain families [7]. Cataloguing all the known interfaces may provide an alternative base for modelling protein tertiary and quaternary structures.

Numerous studies have focused on the characterisation of interfaces using physicochemical properties, shape, packing density, and binding energy [8–16]. The relative orientation between domains or proteins has been studied mostly for particular families of interest [17,18]. Though there are several extensive analyses on the binding orientations [3,19], systematic classifications have been rare [7,20,21]. The paucity of interface classifications is primarily caused by the fact that most interfaces are fragmented and both interacting proteins need to be compared simultaneously. Classic studies on interface characterisation have benefited from a larger and unbiased dataset, resulting in improved prediction methods [22–24].

The potential utility of the representative interface types is diverse. For example, the classification provides a convenient framework for screening common interface motifs shared among homologous and even unrelated folds [21,25]. For docking, the efficiency can greatly improve by restriction to only a few types of known orientations instead of exploring all the possibilities. The improvement of the docking algorithm is facilitated by a more comprehensive benchmark dataset [26]. Along the progress of structural genomics, genomewide modelling of proteins will be realistic with reasonable accuracy in the near future [27–29]. Comparative modelling of interfaces or complexes is expected to follow a similar path. The utility of interface classification will become more significant as building blocks for modelling protein complexes [30].

Our work elaborates and improves the previous work by Kim and colleagues [7] in several ways, which classified the geometry of domain–domain association using patterns of interface residues mapped on the aligned sequences.

Our classification method is primarily based on structural alignments, while the previous work depends on sequence alignments. The classification accuracy significantly improved from 78% recall and 90% precision in [7] to 91% and 92% by a fully structural method. A hybrid approach using both a sequence and structure-based method achieves an accuracy of 83% and 95% with far less computation than the fully structural method. The number of interface types increased by 40% with increasing number of multidomain structures. The utility of the classification is shown by studying hub proteins, gene fusion cases, the conservation of interfaces, and the interface evolution across the three kingdoms of life. All the classifications are online at http://www.scoppi.org with a convenient query environment.

## Results/Discussion

### Domain–Domain Interfaces

Domain interfaces can be defined in various ways, such as the burial of accessible surface area (ASA), interatomic distances, or van der Waals energy, which are shown to be consistent with each other [20]. In this work, interface residues are defined as the residue pairs showing any interatomic distance within 5 Å.

As the interfaces are highly diverse in terms of size, affinity, and shape, no simple criterion is sufficient to discriminate specific and nonspecific interfaces such as crystal-packing artifacts [12,31–33]. In general, interface area (Δ*ASA*) is known as the most significant predictor. According to size, Vajda and Camacho categorised interfaces into large (Δ*ASA* > 2,000 Å), medium (Δ*ASA* < 2,000 Å^2^), and small (Δ*ASA* < 1,400 Å^2^) [34]. In the case of domain interfaces, the interface area can be smaller than protein interfaces because a single interface between proteins may consist of several domain interfaces; thus, the interface size cutoff is set as Δ*ASA* > 800 Å^2^ *.*

The protein structures are taken from the Protein Quaternary Structure (PQS) (http://pqs.ebi.ac.uk) database [35]. Structural Classification of Proteins (SCOP) (http://scop.mrc-lmb.cam.ac.uk) domain definitions are used to group the domains into families and superfamilies [36]. All of the binary domain–domain contacts are checked in the multidomain or multisubunit entries of PQS. In total, more than 70,000 domain interfaces are collected from PQS. The domain pairs are grouped into more than 2,900 distinct SCOP family pairs or 2,000 superfamily pairs.

The interactions are classified into four groups: (1) homo-intra, (2) homo-inter, (3) hetero-intra, and (4) hetero-inter. Homo- or hetero- is assigned depending on whether the interacting domains are from the same family or from different families, respectively. Interaction type intra is assigned to domain pairs from the same chain and inter to pairs from different chains.

### Interface Classification by Face Clustering

Nussinov and colleagues classified interfaces based on common structural features shared among the interfaces from various folds [20,21]. Our method focuses on the diversity of binding orientations between two families, which makes our approach distinct and complementary to Nussinov and colleagues' work. We define a face as a set of interface residues on a single domain contacting with another domain within 5 Å. Accordingly, an interface consists of two interacting faces. Instead of classifying interfaces as a whole, we classify the type of faces in each family and then combine the two types of interacting faces to determine the interface type. The independent clustering of faces makes the classification task highly efficient and straightforward. As the same type of face represents equivalent surfaces of a domain family, the resulting interface type encodes the 3-D geometry of an association. If a domain is in contact with several other domains simultaneously, such a multifaced domain has the same number of faces as the number of its partners and each face is treated independently.

**Figure 1:**
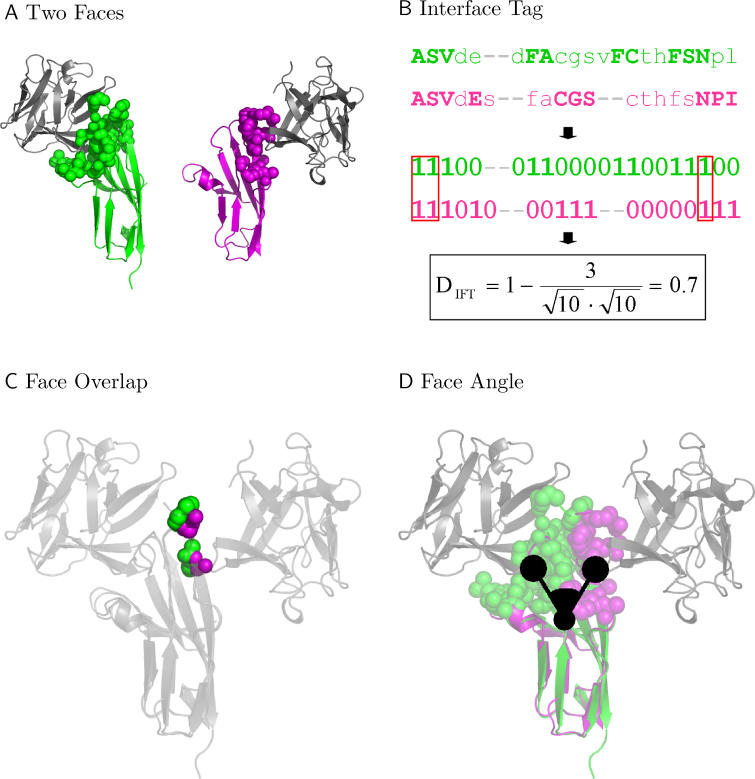
Three Different Features Measuring the Similarity between Two Faces (A) Two faces in I set domain family (green and magenta) interacting with fibroblast growth factor (gray) in different binding orientations. The faces of I set domains are shown in spheres. (B) IFT—the interface residues (uppercase) are mapped by ones and others (lowercase) as zeroes on the aligned sequences. The common patterns of interface residues are outlined with red boxes. The IFTs are simplified just to illustrate the minimal characteristics. In reality, the length of an IFT is the same as its aligned sequence. (C) Face overlap—the interface atoms are highlighted at the intersection of the two faces after superposition of two I set domains. (D) Face angle—the angle between the centres of the two faces and the common centre of the superposed I set domains (see Materials and Methods for details).

To measure the similarity of two faces, we introduce two geometric features—face overlap and face angle—as well as a sequence-based feature—interface tag (IFT; Figure 1). The scale of measurement is set to decrease for more similar faces so that the features are appropriate for clustering. An IFT represents each face and is generated by mapping the face residues onto the aligned sequences. The resulting IFT is a vector consisting of ones and zeros with gaps, where ones represent interface residues and zeros indicate noninterface residues. The distance between two IFTs is measured by *D_IFT_,* where *D_IFT_* = 0 for identical patterns and *D_IFT_* = 1 for faces without common interface residues [7]. The two geometric features are calculated after structural alignment of the two domains. The face overlap distance (*D_O_*) measures the spatial overlap of the interface atoms between two faces. The face angle (*D_A_*) measures the angle between the two centroids of the faces and the common centroid of the two domains. The full descriptions of *D_IFT_, D_O_,* and *D_A_* are given in Materials and Methods.

Hierarchical clustering can be applied at a specified face overlap or face angle cutoff. However, clustering all the faces solely based on geometric features needs enormous computation. Several SCOP families contain more than 1,000–2,000 domains, where each requires about 0.5–2 million structural comparisons. Multifaced domains add more complexity by several folds, as each face should be compared independently. As the known structures are highly redundant, a hybrid approach is applied to reduce the amount of computation. First, faces of highly similar IFT patterns are merged into stage I face clusters at *D_IFT_* < 0.1 to remove redundancy. A representative face is chosen in each stage I face cluster. Second, the representative faces are clustered using the geometric feature of *D_O_* or *D_A_,* resulting in stage II face clusters. The types of the nonrepresentative faces are assigned those of their representatives.

The amount of computation is highly dependent of the size of a family, the length of domains, and the redundancy of data. More than 80% of the families have less than 100 faces, where each family is reasonably computable within several CPU hours, even using a fully structural method. The real bottleneck is the large families with thousands of member domains or faces. There are about 30 families with nearly 1,000 or more faces. As an illustrative example, the ferritin family has 992 domains and is involved in more than 2,400 interactions. There are more than 4,800 faces but only 114 representatives at the redundancy cutoff *D_IFT_* < 0.1. Assuming 1 s for each structural alignment by MultiProt [37], the overlap method requires 4,800 × 4,799/2 comparisons, amounting to approximately 4 mo. The hybrid method requires only 114 × 113/2 comparisons, amounting to approximately 2 h. For all the families in PQS, the hybrid method took 32 CPU days on a 12-node PC cluster, while the overlap method would take more than 3,000 CPU days (Table 1).

**Table 1:**
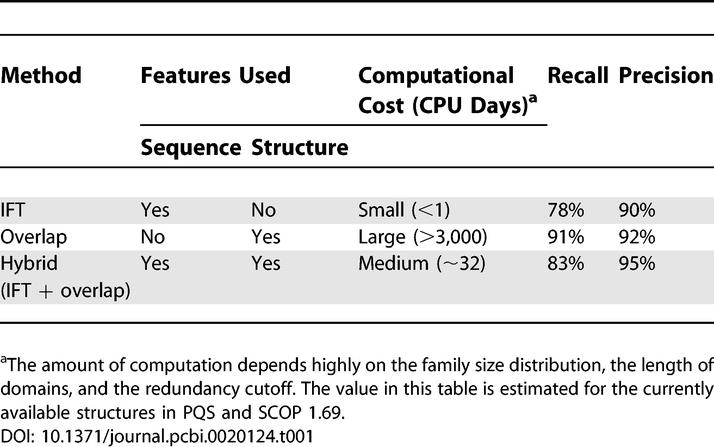
Comparison of Different Classification Methods

After clustering the faces from each family, the face type was denoted as family id:cluster id (i.e., b.34.2.1:03). The types of interfaces were assigned simply by combining the two types of interacting faces. For example, (b.34.2.1:03, d.93.1.1:02,inter) was assigned for the pair of faces b.34.2.1:03 and d.93.1.1:02 from different chains.

### Accuracy of Classification

The classification accuracy was tested using 416 manually classified interfaces between 28 family–family pairs (Table S1). To make the test challenging and rigorous, the family pairs were chosen from family pairs with highly diverse binding orientations, and the interfaces were made nonredundant (NR). On average, the family pairs in the benchmark showed 5.4 distinct binding orientations, or interface types. The benchmark set was made NR by collating domain pairs of similar interface patterns (*D_IFT_* < 0.3) for both faces.

**Figure 2:**
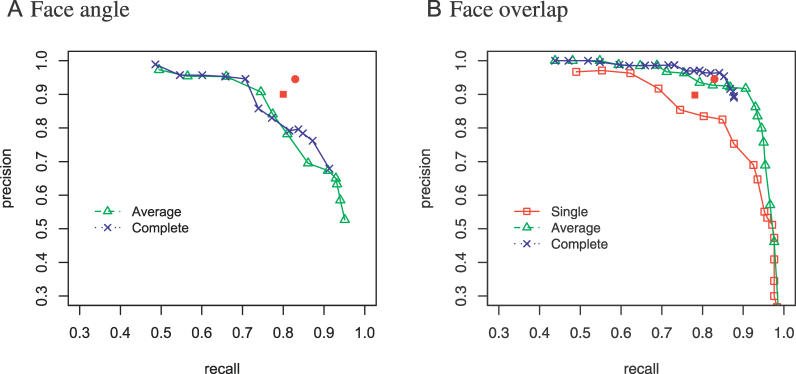
Receiver Operating Characteristic Diagrams of Interface Classification by Hierarchical Clustering Using Different Linkage Methods Single linkage, red empty rectangle; average linkage, green empty triangle; complete linkage, blue cross*.* The recall and the precision by the IFT clustering method (filled rectangle) and by the hybrid method (filled circle) are shown together for comparison. (A) *D_A_*-based classification. (B) *D_O_*-based classification.

A series of hierarchical clustering conditions are tested using *D_A_* ranging from 0°–60° in 5° intervals and *D_O_* ranging from 1%–100% in 5% intervals as cutoffs. The recall and the precision were calculated for each interface type. In Figure 2, the receiver operating characteristic diagrams show the precision and the recall in different clustering conditions. The classification by face overlap consistently shows better accuracy than the face angle method. In comparison with the IFT clustering method [7], the face overlap method shows nearly 10% better recall at the same precision (Figure 2B), while the face angle method shows the lowest accuracy (Figure 2A). Face overlap and IFT clustering use a set of atoms or residues, while the face angle method uses only a single point to represent a face. Accordingly, the face angle method loses the information about shape or volume when the distance is measured. The observed accuracy reflects the order of how well the face definition represents the 3-D shape of each face (overlap > IFT > angle).

*D_O_* = 40% as a cutoff using average linkage was chosen as our clustering condition, which shows 91% recall and 92% precision. In the hybrid procedure, faces with *D_IFT_* < 0.1 were merged as stage I clusters to remove redundancy. The chosen cutoff was set as the stage II clustering condition. The hybrid procedure showed 83% recall and 95% precision, showing a significant improvement from 78% and 90% by IFT clustering alone (Table 1). It suggests that the hybrid procedure achieves an accuracy rate close to that of the purely structure-based method with far less computation. As the classification error tends to be positively correlated with the diversity of interfaces in a family pair, the estimated accuracy is expected to be close to the lower bound [7].

### Interface Diversity

**Figure 3:**
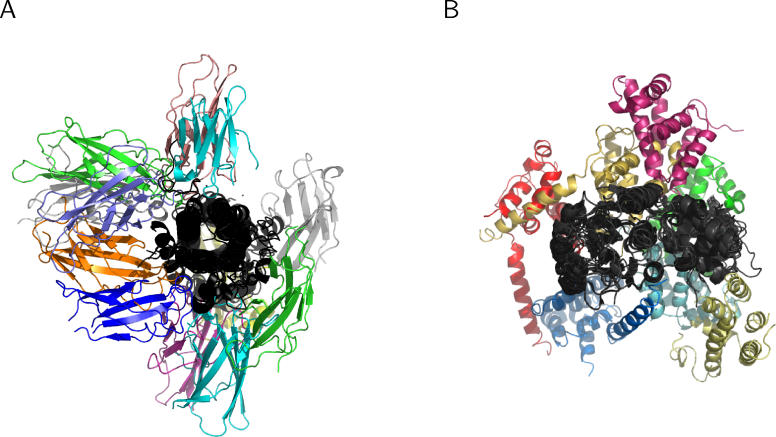
Diverse Modes of Binding Orientations between Interacting Families The domains of one family are superposed at the centre. Some binding orientations are omitted for a clear view. (A) Long-chain cytokines (centre) and fibronectin type III (peripheral). (B) Extended AAA–ATPase domain (centre) and DNA polymerase III clamp loader subunits, C-terminal domain (peripheral).

**Figure 4:**
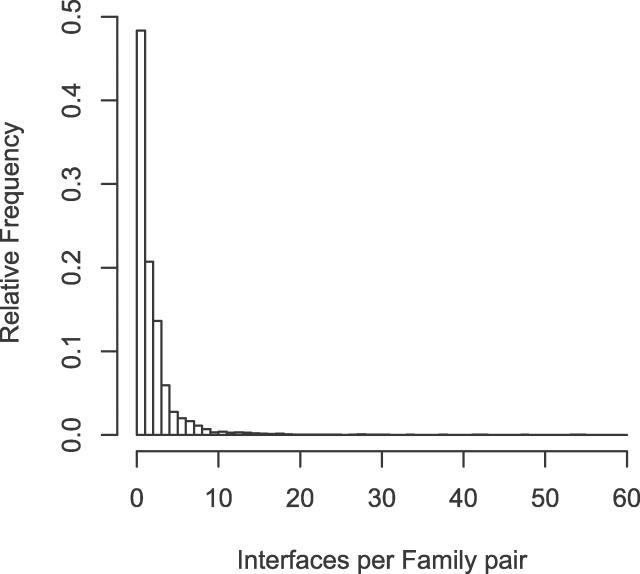
The Number of Different Interface Types between a Pair of Families

As some families interact in highly diverse orientations (Figure 3), knowing the number of different interaction modes between two families is critical to model protein complexes using known structures. We checked the extent of multiple interface development between the family pairs in our dataset. The result shows that there are 1.2–2.2 different types of interfaces per family–family pair depending on the interaction category (Table 2). About 60% of the family pairs associate in a unique orientation, and the remaining 40% show multiple types of interfaces (Figure 4). In intermolecular interfaces, multiple interfaces in homodimers (46%) are about two times more frequent than heterodimers (24%). It is expected by frequent occurrence of homo-oligomeric proteins or complexes in PQS that any oligomer of three or more components should form multiple interface types. Interestingly, 17% of the intra-type family pairs are shown to have multiple interfaces although they mostly appear in the same N-to-C sequence order. The diversity of binding orientations in intra-type family pairs is caused generally by structural flexibility at the linker region between two domains.

**Table 2:**
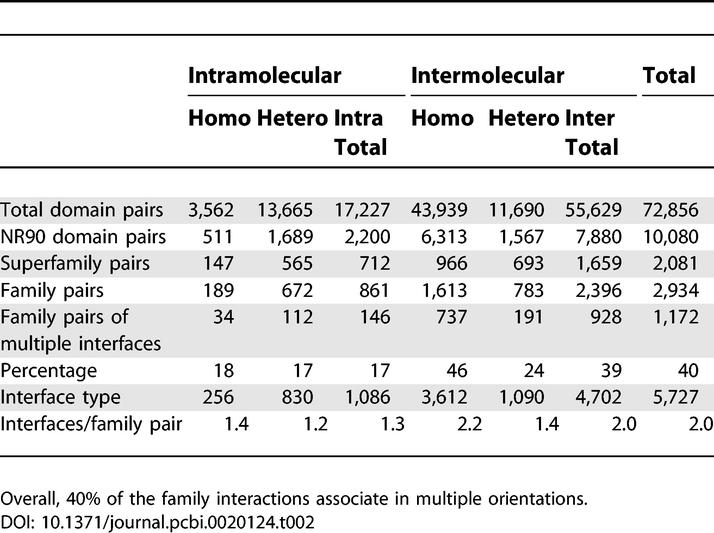
Summary of the Interface Classification

**Figure 5:**
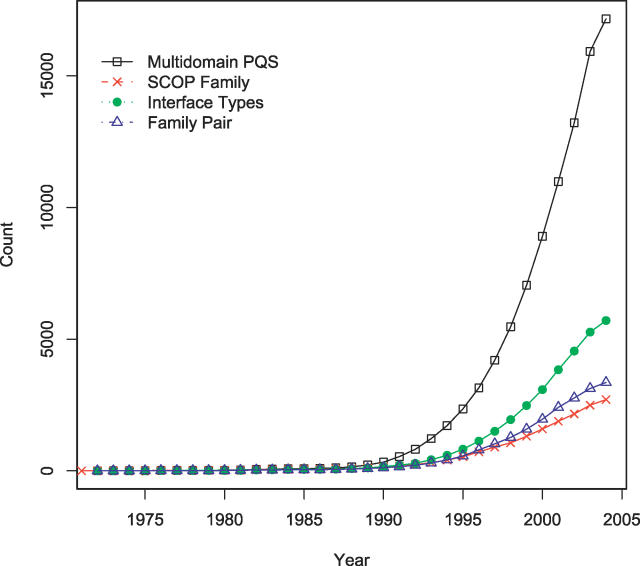
The Growth of Structures, SCOP Family, Family Pairs, and Interface Types

The annual growth of interface diversity was checked along with the number of multidomain structures, families, and family pairs (Figure 5). Multidomain structures grew rapidly after the 1990s because of structural genomics programs and the technical advance of X-ray crystallography and nuclear magnetic resonance. Nearly 90% of the interface types became available only in the last 10 y, and 50% in the last 5 y. This suggests that in many of the past interface analyses, the scoring systems for docking and the prediction methods for binding sites were based on a small fraction of interface types currently available. The number of interfaces grew quickly until recently, suggesting that many types of unknown interfaces still remain to be discovered, as suggested by Aloy et al. [30]. In comparison with the previous work [7], our work shows about 40% increase of domain interfaces in 14 mo between the two SCOP versions (1.65 and 1.69). The number of distinct family pairs and interface types also increased at a similar rate. It is notable that the ratio of hetero-inter among the total interface types increased by 13% from the previous 16.8% to 19.0%, while the portion of homo-intra dropped by 19% from 5.5% to 4.5%. This trend suggests that structures of multichain complexes have grown more rapidly than single-chain or homo-oligomeric ones.

### Hub Proteins Have Many Faces

**Figure 6:**
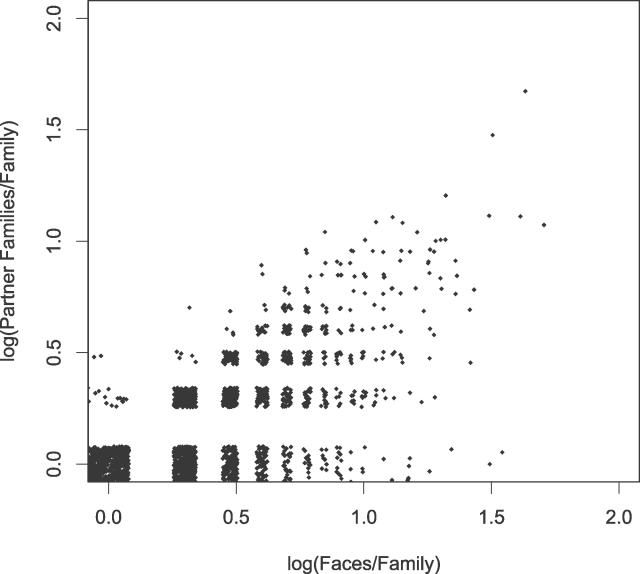
The Relationship between the Number of Partner Families and the Number of Faces per Family The datapoints are jittered slightly to show the points of the same value.

Currently, much research is devoted to study protein interaction networks as a whole, and hub proteins in particular. Hub proteins are a set of proteins highly connected to many other proteins in a network. Our classification can shed light onto the relationship between the number of partners (*N_Partner_*) and the number of faces of a family (*N_Face_*)*. N_Partner_* and *N_Face_* show a positive correlation of 0.66. Most families with multiple partners have multiple face types. The number of face types is generally similar to or higher than the number of partner families, showing a lower triangular pattern in Figure 6. In general, a family develops one or more distinct faces for each partner, such as the G-proteins family, which has 43 faces for 47 partner families. In one extreme, multiple faces are a result of developing multiple interaction modes between two families, such as the long-chain cytokines family, which has 12 faces for two partner families (Figure 3A). On the other end, a family uses an equivalent surface for multiple partners, such as the PUA domain family, with only two face types for five different partner families.

Besides the highly connected families in our classification, we consider known hub proteins identified by other experimental methods and try to relate the number of partners to the number of faces. Dunker and colleagues suggest protein disorder as a mechanism for hub proteins to bind multiple, structurally diverse partners [38]. They classified 14 known hub proteins into three classes according to the ratio of disordered regions, which are indicated here as (mostly) disordered, intermediate, and (mostly) ordered (Table 3). We checked whether the 14 hub proteins tended to have many faces in our classification after assigning SCOP domains using PSI-BLAST. A single SCOP domain was assigned for six hub proteins, and two SCOP domains for other two proteins. The ratio of domain assigned regions varied from 8% to 100% of the total length of each protein. The remaining six hubs were not assigned any SCOP domains, which were all in the disordered or intermediate classes, reflecting the difficulty of crystallization for disordered proteins. Interestingly, three out of the four ordered hub proteins had significantly more faces than expected (*p* < 0.05). The 14-3-3′ζ protein is an exception, with only two faces. However, the 14-3-3′ζ protein is likely to have more faces because there are also only two partner families of 14-3-3′ζ: itself and the N-acetyl transferase family. In the intermediate class, the estrogen receptor α has 20 faces, but 18 of them are for homodimeric interactions. In the two other intermediate proteins and the one disordered protein, the assigned SCOP domains had a comparable number of faces to the number of partner families. For example, there were eight faces for seven partner families in the RING finger domain of BRCA1, three faces for three partners in p53, and six faces for four partners in the serine/threonine phosphatase domain of clacineurin subunit A.

**Table 3:**
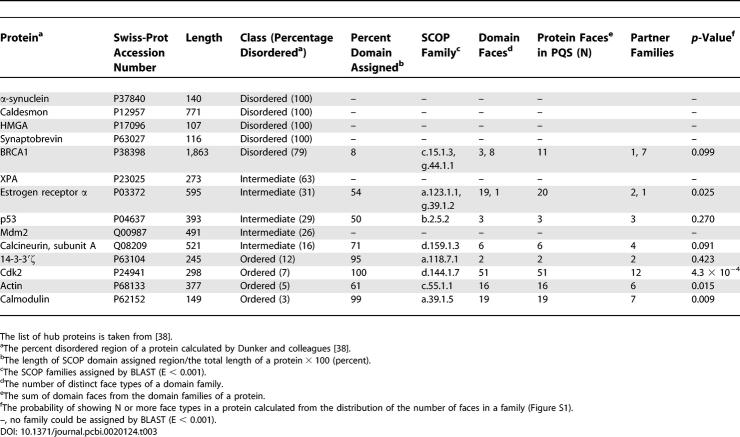
The Face Diversity of Known Hub Proteins

Our results show that the three ordered hub proteins (cdk2, actin, calmodulin) develop significantly higher number of faces than expected. In the remaining five proteins, seven SCOP families were assigned, and five of them had nearly one face per partner. Obviously, current structural data do not contain all the interactions of hub proteins. However, the face diversity of the eight hub proteins strongly suggests that hub proteins tend to use distinct surface regions for different partners.

In the course of evolution, hub proteins may duplicate, differentiate, and develop novel functions with a different set of interaction partners. Such a family needs to continuously develop new interactions and break existing interactions. By having distinct faces, hub proteins may avoid crosstalk or competition between different interactions and allow modular evolution of interfaces. From our observation on the eight hub proteins, we postulate that a face may serve as an independent evolutionary unit to provide a physical basis for complex wiring around hub proteins in an interaction network.

The multifaced nature of hub proteins provides an opportunity to dissect the role of each interaction with diverse partners. Functional genomics approaches such as gene knockout or RNA interference remove a whole gene, thus eliminating all the connections around. For hub proteins, this knockout approach is complicated to interpret because many pathways or functions are influenced simultaneously. In contrast, the engineering of each face may specifically interfere with a certain type of interaction without influencing others. Conversely, the mutations causing a certain phenotype can be traced among a series of mutants on random positions, leading to the identification of specific interactions or partners responsible for the phenotype. The design of novel interfaces has already been achieved successfully for several proteins, including calmodulin and PDZ domain, as reviewed by Kortemme and Baker [39].

### Genuine Gene Fusions

**Figure 7:**
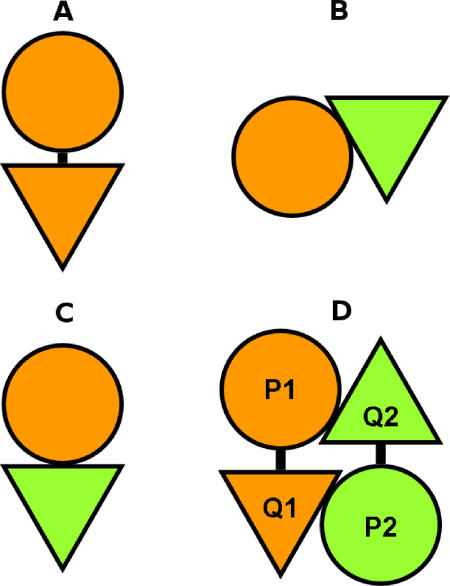
A Schematic Diagram of Genuine and Nongenuine Fusions, Where Two Domains Exist Both in a Fused Form and as Separate Proteins (A) Fused form. (B,C) Separate proteins. A genuine gene fusion conserves the binding orientation (A and C) but nongenuine fusion does not (A and B). A spurious gene fusion case can be found in a homodimer of a multidomain protein, where *P_1_Q_1_* and *P_2_Q_2_* are the same protein of identical sequence (D).

Conventionally, gene fusions or domain fusions are identified by sequence similarity search for two separate proteins in one organism appearing as a single homologous fusion protein in another organism. However, there has been no work to check how often the binding orientation is conserved. Here, we define genuine gene (domain) fusion as the subset of conventional gene fusion cases that associates in the same orientation in both the separate and the fused forms, and the rest as nongenuine gene (domain) fusion (Figure 7).

Based on our classification, the genuine gene fusion cases are screened systematically as the interface type appearing as both “intra” and “inter.” The type of interaction is assigned as “inter” if the Protein Data Bank (PDB) chain IDs are different, and as “intra” if the chain IDs are the same. However, some proteins may be fragmented by engineering and post-translational modification, resulting in different PQS chain IDs. Alternatively, different proteins may be fused by an artificial linker, giving the same chain ID in PQS. For example, α-amylase from Bacillus licheniformis is fragmented into two chains by trace amounts of Glu-C peptidase included during the sample preparation [40]. Because gene fusion should be checked using the whole chain (protein), it adds more complexity that interfaces are classified at the domain level. False gene fusions can be found when a chain consists of multiple domains and the chain forms homo-oligomers in PQS data. For example (see Figure 7D), if a chain consisting of two domains (*P, Q*) forms a homodimer (*P_1_Q_ 1_, P_2_ Q _2_*) that has interfaces between *P_1_* and *Q_2_* and between *P_ 2_* and *Q_1_,* two hetero-inter domain–domain interfaces are formed from one chain homodimer. Obviously, a chain homodimer has no relevance to gene fusion because the two chains originated from the same gene. The spurious gene fusion cases were filtered out using the link between PDB chain and UniProt as described in Materials and Methods.

Only a small fraction of family pairs appeared both in fused and separate forms in PQS (Table 4). In screening nongenuine fusions, we also filtered out PQS entries containing both intra- and inter-type interfaces of a given family pair, avoiding spurious gene fusion from chain homo-oligomers. Therefore, there could be more nongenuine fusion cases in our dataset. To our surprise, two-thirds of gene fusions associate in the same orientation, while the remaining one-third interacts differently. As interfaces in PQS represent only a small fraction of diversity in nature, some of the nongenuine cases could turn out to be genuine if the same type of interfaces were found in either separate or fused form. Nevertheless, the finding that at least one-third of gene fusions interact in different orientations has significant implications in structure analysis. For example, the proteins are often fragmented or fused to facilitate crystallisation or to increase stability. The native structure may not be the same as the artificially fused or fragmented form, which could lead to misinterpretation of the mechanism of protein function. The lists of genuine and nongenuine fusion cases are in Tables S2 and S3.

**Table 4:**
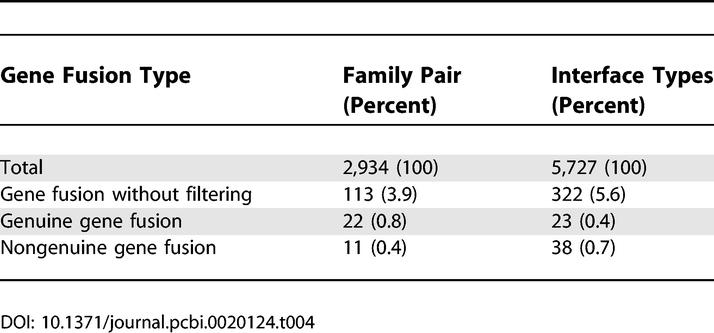
Domain Fusion Cases in PQS, Where Two-Thirds of the Gene Fusions Are Genuine and One-Third Are Nongenuine

**Figure 8:**
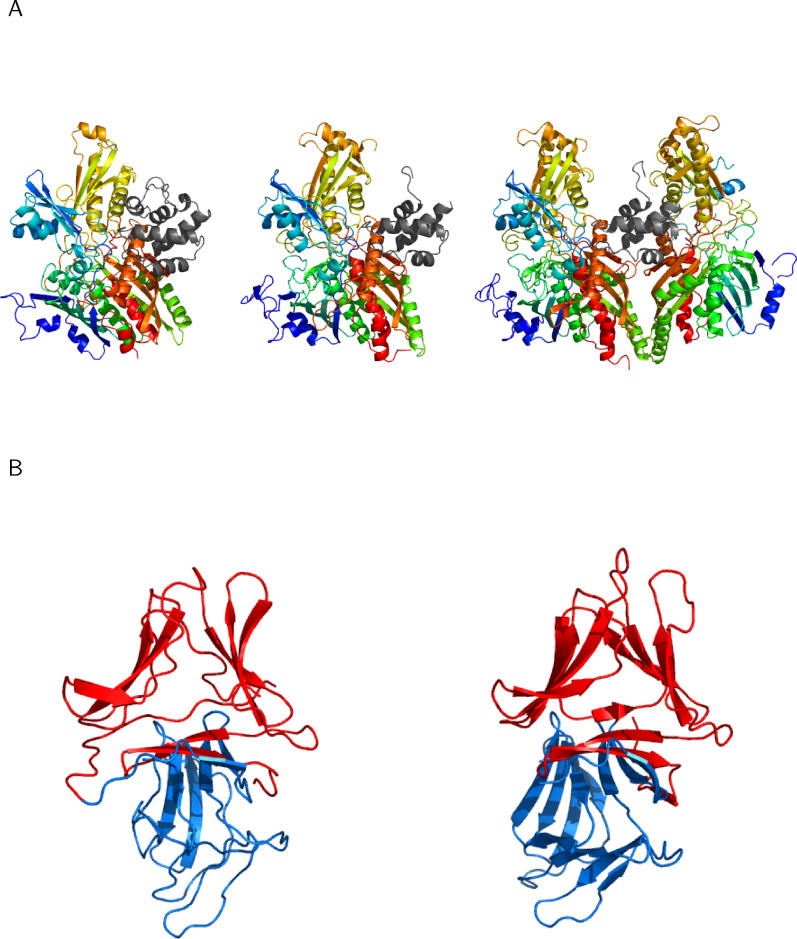
Examples of Genuine Gene Fusion (A) Heterodimeric interfaces between CO dehydrogenase ISP C-domain–like family (gray) and molybdenum cofactorbinding domain family (rainbow). The two families show conserved binding orientation. Left: fused domain pair from aldehyde oxidoreductase of Desulfovibrio desulfuricans. Centre: two separate molecules in CO dehydrogenase of Hydrogenophaga pseudoflava. Right: two CO dehydrogenase ISP C-domains and one molybdenum cofactor-binding domain in CO dehydrogenase from Oligotropha carboxidovorans showing one conserved and the other variable interface type. (B) Homodimeric interfaces between two alpha-D-mannose–specific plant lectin families. Left, fused domain pair of Scilla campanulata agglutinin. Right, two separate molecules in Allium sativum lectin.

One example of gene fusion is the CO dehydrogenase ISP C-domain–like family and molybdenum cofactor-binding domain family pair (Figure 8A). In aldehyde oxidoreductase of *Desulfovibrio desulfuricans,* the two domains are fused in a single protein, while they exist as two separate proteins in CO dehydrogenase of Hydrogenophaga pseudoflava and *Oligotropha carboxidovorans.* The binding orientation is conserved between the two enzymes of D. desulfuricans and *H. pseudoflava.* However, the two domains in CO dehydrogenase of O. carboxidovorans also form another type of interface. It is interesting that the latter two enzymes develop divergent interfaces showing the same molecular function (carbon monoxide dehydrogenase), while the first two have the same interface in spite of slightly different molecular functions (carbon monoxide dehydrogenase and carboxylate reductase). The alpha-D-mannose–specific plant lectin family is another example of genuine gene fusion. This family appears both as a fused homodimer, *Scilla campanulata* agglutinin in *S. campanulata,* and as two separate proteins, lectins, in Allium sativum (Figure 8B).

**Figure 9:**
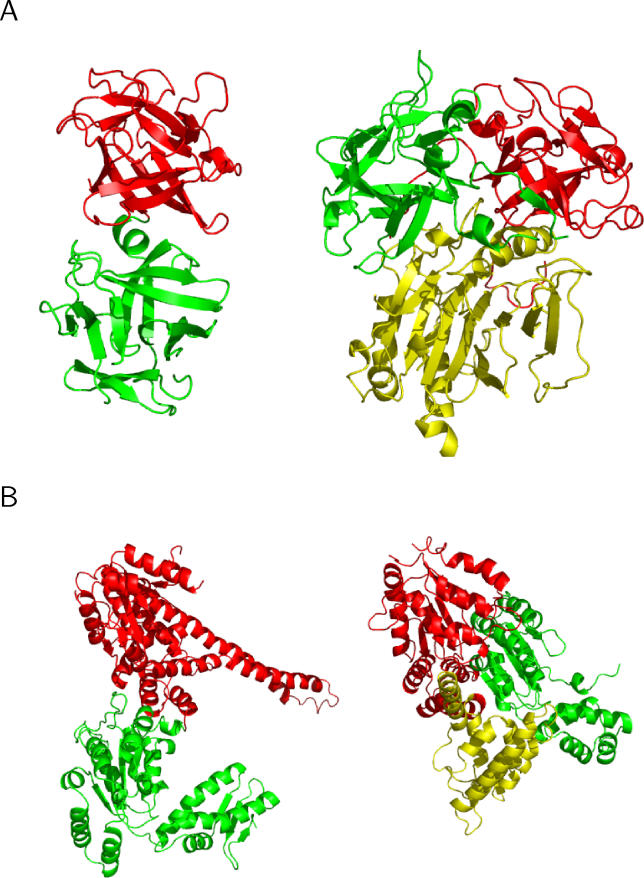
Examples of Nongenuine Gene Fusion, Where a Domain Pair Associates in Different Binding Orientations between Intra- and Inter-Types, Where an Additional Interaction Partner Occupies the Same Face of One Domain in Inter-Type Intra-types are shown on the left, and inter-types are shown on the right. The domains in red are all in parallel orientations. (A) Homodimers of ricin B-like domains (red and green) and a DNase I-like domain (yellow) in haemagglutinin component (HA1) of the progenitor toxin from Clostridium botulinum (left), and Haemophilus ducreyi cytolethal distending toxin (right). Whereas the ricin-like domains of the haemagglutinin component (HA1) of Clostridium botulinum progenitor toxin (left) function by binding of carbohydrates [61], a different association of these domains in the Haemophilus ducreyi holotoxin (right) gives rise to a completely different function. Here, both domains contribute to the formation of a groove that acts as a potential peptide binding site to initiate endocytosis of the holotoxin complex [62]. (B) Homodimers of extended AAATPase domain family (red and green) and a DNA polymerase III clamp loader subunits, C-terminal domain (yellow). The AAA-ATPase domains are known to couple ATP binding/hydrolysis to protein assembly/disassembly [63]. AAATPase domains associate in different orientations in ClpB protein, a molecular chaperone disaggregating stress-damaged proteins (left) [64] and in a DNA clamp loader complex (right). An additional domain, DNA polymerase III clamp loader subunits, C-terminal domain (yellow), is present and unique to clamp loaders (right) [65].

In nongenuine fusions, there are cases where a face in one interface type is occupied by an additional interaction partner in the second interface type, resulting in an alternative binding orientation. That is, a family pair A,B shows alternative orientations (A:a,B:b) and (A:x,B:y) because a third partner C:c occupies the face of family A (A:a) using an interface type (A:a,C:c). This partner exchange is observed in two of the 11 family pairs of nongenuine fusions. Some domain pairs may function independently without geometric contraint, resulting in various binding orientations as in SH3 and SH2 [3]. The partner exchange is shown as one of the causes, and the examples of the two family pairs are shown in Figure 9.

### Conservation of Interface Residues

The residues at protein interfaces are considered conserved [13,41] because of the evolutionary constraint to maintain interactions. The conservation of interfaces is used for the prediction of binding sites [42,43]. However, it is also argued that the interfaces are conserved only marginally more than the other sequences [44,45]. In studying interface conservation, homologous sequences are generally added without considering the possibility of multiple binding orientations or partners, which may complicate the results.

**Figure 10:**
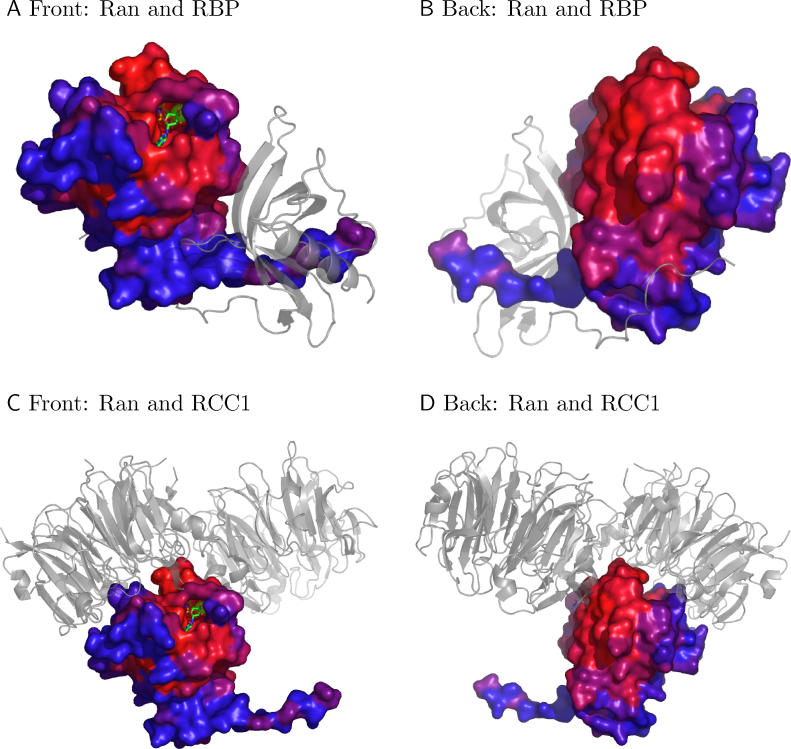
The Conservation of Residues on the Surface of Ran GTPase The conservation score is derived from Consurf–HSSP and is color-coded, with blue being most variable and red most conserved [42]. (A,B) Front and back of the less-conserved interfaces between Ran GTPase and Ran-binding protein (RBP, gray) in Ran-binding domain complexed with Ran bound to a GTP analogue. (C,D) Front and back of the same Ran GTPase interacting with the RCC1 (gray) protein in the guanine nucleotide exchange on Ran by the regulator of chromosome condensation (RCC1). The highly conserved, prominent bulge protrudes the cleft between the homodimer of RCC1 proteins. The GTP-binding pocket of Ran GTPase is also well-conserved.

We took an example from the Ran family in Caffrey and colleagues' analysis [45], where the reason for poor conservation was not clear, but where the authors suspected the existence of additional interfaces. Indeed, another interface with the regulator of chromosome condensation (RCC1) domain was found in our classification. Figure 10 clearly shows that the interface of the Ran family with the Ran-binding domain is poorly conserved (Figure 10A and 10B), while the interface with RCC1 as well as the GTP-binding pocket show strong conservation (Figure 10C and 10D). The additional interface with the RCC1 domain was not included in the analysis, although the authors suggested a correct hypothesis and the data were already available in PDB (guanine nucleotide exchange on Ran by the regulator of chromosome condensation). It suggests that our comprehensive classification may be useful in other kinds of interface studies.

### Ancient Interfaces Are Dominated by Symmetric Homodimers

How did different interface types evolve, and how many of them are common among species or lineage-specific? The questions of the evolutionary history and the taxonomic distribution of interfaces are highly interesting but difficult to answer due to the bias and the low coverage of structures available. Gene duplication and differentiation are an important mechanism to develop more complex protein functions in higher organisms. The direction of interface evolution tends to be from symmetric to asymmetric homodimers and heterodimers [46]. Here, we perform a preliminary analysis on the taxonomic distribution of interactions and interfaces in the three major kingdoms of life—archea, bacteria, and eukaryotes.

In terms of family pairs, archea have the most overlap with other kingdoms, probably because archea are the most primitive form of life (Figure S2). There are 75 family pairs that have member domain pairs from all the kingdoms. For a statistical interpretation, 23 family pairs were selected out of the common 75 family pairs, each with a taxonomic diversity of ten or more species. These core 23 family pairs consist of 127 inter- and ten intra-type interfaces originating from 160 species.

**Figure 11:**
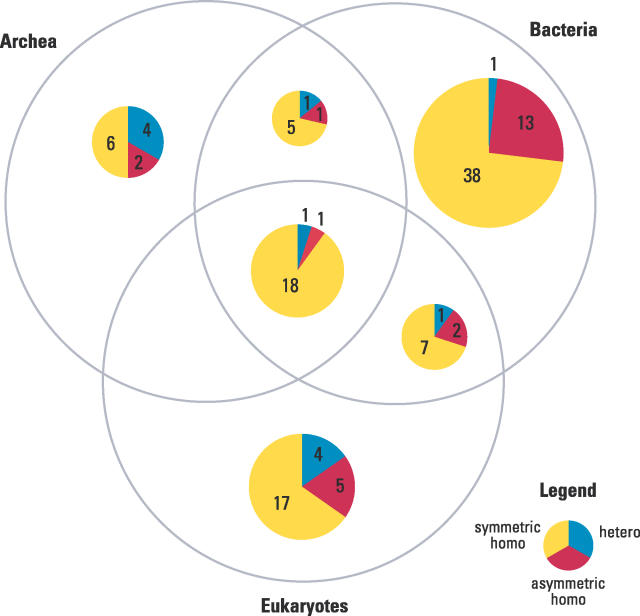
The Distribution of 127 Interfaces and Their Categories from 23 Family Pairs Common to All Three Kingdoms and Having Ten or More Species Diversity The category of the interfaces are divided as homo and hetero. Sym-homo (symmetric homodimer) associates using the faces of the same type and asym-homo (asymmetric homodimer) using the faces of different types. The 20 common or ancient interfaces are mostly symmetric homodimeric.

It was assumed that an interface type is ancient if it is common to all three kingdoms. The taxonomic distribution of the 127 inter-type interfaces is shown in Figure 11. Interestingly, the common or ancient 20 interfaces all belong to symmetric homodimers, with only two exceptions (one hetero and one asymmetric homo). Asymmetric homo- and hetero-types are enriched in the lineage-specific category, though not as strongly as in the ancient category. This observation supports the trend of interface evolution from symmetric to asymmetric or hetero. In contrast to ancient types, the identification of lineage-specific types is always ambiguous because the structures may simply not be available across multiple kingdoms. Here, 90 of the 127 interfaces were found to be lineage-specific to a single kingdom. However, only five of these were estimated significantly lineage-specific (*p* < 0.01): four symmetric and one asymmetric homo-types. The significance was calculated as the probability of sampling *N* times only the species of the single kingdom out of the total species found in the corresponding family pair, where *N* is the number of member domain pairs of the interface type. In the ten intra-type interfaces, there were one symmetric, two asymmetric, and five hetero-types, where five hetero-types were ancient (unpublished data). None of the lineage-specific interfaces were significant because of the small amount of data. Overall, this analysis supports the hypothesis that ancient interactions are symmetric homodimers.

### Database of Domain Interfaces

The classification of domain interfaces is available online as part of the SCOPPI database [47] at http://www.scoppi.org. Here, the presence of at least five distinct residue–residue contacts within 5 Å rather than a minimal Δ*ASA* size was used as inclusion criterion for an interface [48]. While this may result in small and nonspecific interfaces, we leave it to the user to choose from the comprehensive dataset and allow filtering according to Δ*ASA*.

SCOPPI can be queried for a SCOP family, superfamily, one or several PDB identifiers, or a keyword. Various interface characteristics such as number, type, and position of interacting amino acids; conservation; interface size; and permanent or transient nature of the interaction are provided. In addition, screenshots are available for every interface and its participating domains.

### Conclusion

Multidomain structures have been rapidly increasing since the 1990s. We classified virtually all the domain interfaces found in known structures, resulting in nearly 6,000 distinct interface types. Purely structure-based classification achieves the best accuracy of 91% recall and 92% precision, but requires enormous computation. Our hybrid procedure achieves a similar accuracy of 83% recall and 95% precision, while saving the amount of computation 100-fold. The representative set of interfaces is available at various degrees of redundancy cutoff (50%–100%).

The interfaces are shown to be highly diverse even between homologous pairs of proteins. In our analysis, nearly 40% of families associate in multiple orientations. Some family pairs form extremely diverse interfaces, as shown in the cytokine/fibronectin pair and the AAA-ATPase/DNA polymerase III clamp loader subunits pair (Figure 3). The result suggests that a careful consideration of alternative interfaces will be necessary in modelling tertiary or quaternary structures using known interfaces. In terms of computational complexity, the advantages of using interface classification over combinatorial docking parallels homology modelling over ab initio prediction of protein folding. Assuming three candidate docking solutions, 3^(20−1)^ ≈ 1.2 × 10^9^ conformations need to be explored for modelling complexes of 20 subunits by combinatorial docking. With interface classification, only 3^(20−1) × 0.4^ ≈ 4,200 conformations are to be explored, assuming 40% of the family pairs have, on average, three types of interfaces. Although the known interfaces represent only a small fraction in nature, interface modelling is expected to play a critical role in combination with other experimental and computational methods [49].

Our analysis sheds light onto diverse aspects of interface geometry and evolution. 1) Promiscuous interactions. Hub proteins are shown to interact with various partners using many distinct faces, suggesting face as a module for flexible wiring around hub proteins. In general, the number of partners and faces correlates. 2) Gene fusion. To our knowledge, we provide the first comprehensive screen for gene fusion cases of known structure and check their interaction geometry. About two-thirds of gene fusions are shown to conserve their binding orientation. In at least one-third of the cases, fusion or fission resulted in different geometry. The natural gene fusion cases may provide a good clue in designing chimeric proteins for research, industry, or medicine. 3) Interface conservation. The apparently poor conservation of interfaces (e.g., in the Ran domain family) is due to the diversity of interactions and partners, suggesting any evolutionary interpretation can be affected similarly such as coevolution [50] or binding-site analysis [51,52]. 4) Ancient interfaces. The ancient interfaces common to archea, bacteria, and eukaryotes are shown to be mostly symmetric homodimers. This suggests that asymmetric and hetero interactions evolved from these symmetric homodimers.

The above-detailed results have larger implications: a protein domain is regarded currently as a basic unit of protein structure and function [53–55]. Our results suggest that faces are equally important units, which is especially important when considering interactions and evolution. The diversity of interface types is rapidly increasing by selecting structure targets with much less bias than before [28]. Although it is a daunting task to determine the structures of all the representative interfaces in nature [30,49], we observe that more than 90% of the current 6,000 interface types became available only in the last ten years, and expect even more interfaces to accumulate in the next ten years (Figure 5). The predictions on interfaces and docking algorithms are expected to improve as a larger and unbiased set of interfaces is used. Efforts will continue to understand the physical basis of the organisation of interaction network and its evolution [56].

## Materials and Methods

### Generation of domain–domain interface set

The protein coordinates are taken from PQS (http://pqs.ebi.ac.uk) [35]. SCOP version 1.69 is used to define domains and to group them into families and superfamilies [36].

The Δ*ASA* is calculated as Δ*ASA* = *ASA_A_ +* Δ*ASA_B_* − Δ*ASA_AB_*, where *ASA_A_* and *ASA_B_* are the ASAs of the two isolated domains and *ASA_AB_* is that of the bound form. The *ASA* is calculated by NACCESS, implementing the Lee and Richards algorithm [57].

Both face angle and face overlap were computed by Python scripts using PyMOL (http://www.pymol.org). Out of the 2,403 SCOP families (version 1.69), a few families were excluded because of either weak biological interest or incompatibility with PyMOL script or MultiProt. For example, most domains of collagen-like peptides family have sequences that are too short for alignment by MultiProt. The immunoglobulin family (V set domains [antibody variable domain-like]) was also excluded because the binding partners can be any foreign proteins. The unclassified families were less than 0.5% of the total families; thus, the influence on the result was expected be marginal.

### IFT and IFT distance

The IFTs of each family were generated in a similar way as in the previous work [7]. The face residues were defined as the residues containing at least one atom contacting with the other domain within 5 Å of distance cutoff. The IFT was generated by mapping the face residues onto the aligned sequences by MUSCLE [58] for each family. After the alignment, the interface residues were converted to ones and noninterface residues to zeros. The resulting IFT became a vector of ones and zeros with gaps (e.g., 10-01110-00 for an aligned sequence, Ms-aHCWk-im [interface residues in uppercase and noninterface residues in lowercase]). As all the domain sequences are aligned simultaneously in a family, the lengths of IFTs were the same within the same family.

The difference of IFT patterns was measured as the cosine distance of the two IFT vectors, where the positions containing gaps on either of the two IFTs are ignored.

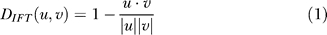
As the elements of each vector consist of only ones and zeros, the distance becomes zero between identical IFT pairs and one between IFT pairs without any common interface residue.

### Face overlap distance and face angle

There are two geometric features—face overlap distance and face angle—to measure the distinctiveness between two faces. Both features were calculated after the superposition of two domains in a family with MultiProt [37].

*D_O_* was measured as

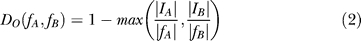
| *I_A_* | , | *I_B_* | are the number of intersection atoms and | *f_A_* | , | *f_B_* | are the total number of atoms in each face, respectively. The intersection atoms of one face are defined as the atoms within 3 Å from the other face atoms. Accordingly, a face fully subsumed by the other yields *D_O_* = 0.

*D_A_* measures the angle between the two centroids of the two faces and the common centroid of the two domains. The centroid was determined by using the *C_α_* carbons of the face residues or the domain residues for computational efficiency.

### Measurement of interface classification accuracy

The accuracy was measured by comparing a test (*T*) and a reference (*R*) classification on a set of interfaces. The different interface types are denoted as *R_i_* and *T_j_,* respectively. The number of domain pairs belonging to each interface type is given as | *R_i_* | and | *T_j_* |, respectively. Then, each reference classification (*R_i_*) was mapped to one of the test classification (*T_j_*)*,* which maximizes | *R_i_* ∩ *T_j_ |*, the number of common domain pairs between the two classifications.

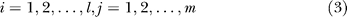


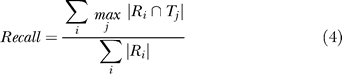
Once the mapping of *R_i_* to *T_j_* (*i* → *j*) was done to maximize recall, precision was calculated as follows:

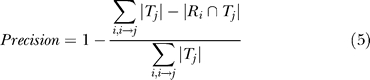
The recall measures the ratio of the interfaces of the same type, which correctly grouped together by the test classification. The precision measures how much the test classification erroneously merged different types of interfaces into the same group. An ideal classification would result in recall = 1 and precision = 1.

### NR set of interfaces

A series of NR interface sets was generated at different sequence identity thresholds from 50% to 100% with a 5% interval. First, a set of NR sequences was generated at each threshold for each SCOP family using CD-HIT [59]. Then, all the domains were represented by the NR domains at a given threshold. Second, NR interfaces were generated by collating the domain pairs with the same pair of representative domains within the same interface type. Intermolecular and intramolecular interfaces were not merged but treated separately.

### Filtering spurious gene fusions

Spurious gene fusion cases were filtered out using the links between PDB chain IDs and UniProt IDs provided by the Macromolecular Structure Database [60]. In gene fusion, the two domains should point to different UniProt IDs for inter-type and to the same UniProt ID for intra-type. The interfaces were excluded in the gene fusion analysis when: 1) two domains of the same chain ID points to different UniProt entries; and 2) two domains of different chain IDs point to the same UniProt ID. The former includes fused proteins by artificial linkers. The latter may contain fragmented proteins by engineering or chain homodimers. More than 90% of the PDB entries have links to UniProt for at least one chain in the interface dataset.

## Supporting Information

Figure S1 Probability Distribution of the Number of Distinct Face Types per Protein The number of faces for a 1 domain protein is calculated from the interface classification. The other distributions for 2, 3, 4 domain proteins are derived from that of 1 domain protein. For single domain proteins, we observe that more than 30% of the domain families display only one face, whereas the protein kinase catalytic subunit family (d.144.1.7) shows the most face diversity of 51 face types. As the number of domains in a protein increases, the distribution shifts towards a higher face average, since its domain faces add up. The average number of faces for a 1, 2, 3, 4 domain protein is 3.2, 6.4, 9.6, 12.8, respectively.
(30 KB PDF)

Figure S2 Taxonomic Distribution of Family Pairs in PQS across Three Kingdoms of Life— Archea, Bacteria, and Eukaryotes (29 KB PDF)

Table S1 Benchmark Interaction Interfaces The benchmark dataset contains a hand-curated set of interaction interfaces, which are particularly difficult to classify.
(19 KB TXT)

Table S2 Genuine Gene Fusion Genuine gene fusion cases, in which the binding orientation of the fused and nonfused domains is preserved.
(30 KB TXT)

Table S3 Nongenuine Gene Fusion Nongenuine gene fusion cases, in which the binding orientation of the fused and nonfused domains is not preserved.
(41 KB TXT)

### Accession Numbers

The SCOP (http://scop.mrc-lmb.cam.ac.uk) accession numbers for the domain families mentioned in this paper are 14-3-3 protein (a.118.7.1), AAA–ATPase domain (c.37.1.20), actin/HSP70 (c.55.1.1), breast cancer associated protein, BRCA1 (c.15.1.3), calmodulin-like (a.39.1.5), protein kinases, catalytic subunit (d.144.1.7), CO dehydrogenase ISP C-domain–like (a.56.1.1), collagen-like peptides (k.3.1.1), DNA polymerase III clamp loader subunits, C-terminal domain (a.80.1.1), ferritin (a.25.1.1), DNase I-like domain (d.151.1.1), fibronectin type III (b.1.2.1), G proteins (c.37.1.8), long-chain cytokines (a.26.1.1), molybdenum cofactorbinding domain (d.133.1.1), nuclear receptor (g.39.1.2), nuclear receptor ligand-binding domain (a.123.1.1), p53 DNA-binding domain-like (b.2.5.2), protein serine/threonine phosphatase (d.159.1.3), PUA domain (b.122.1.1), ricin B-like domains (b.42.2.1), RING finger domain, C3HC4 (g.44.1.1), V set domains (antibody variable domain-like) (b.1.1.1).

The Protein Data Bank (http://www.pdb.org) accession numbers for the structures mentioned in this paper are α-amylase from Bacillus licheniformis (1bli), aldehyde oxidoreductase of Desulfovibrio desulfuricans (1dgj), Allium sativum lectin (1kj1), CO dehydrogenase of Hydrogenophaga pseudoflava (1ffu), DNA clamp loader complex (1sxj), fused domain pair of Scilla campanulata agglutinin (1dlp), guanine nucleotide exchange on Ran by the regulator of chromosome condensation (RCC1) (1i2m), haemagglutinin component (HA1) of the progenitor toxin from Clostridium botulinum (1qxm), Haemophilus ducreyi cytolethal distending toxin (1sr4), ClpB protein (1qvr), molybdenum cofactor-binding domain in CO dehydrogenase from Oligotropha carboxidovorans (1n60), Ran-binding domain complexed with Ran bound to a GTP analogue (1rrp).

The Enzyme Classification numbers (http://www.expasy.ch/enzyme) for the enzymes mentioned in this paper are carbon monoxide dehydrogenase (1.2.99.2) and carboxylate reductase (1.2.99.6).

## Abbreviations

ASA: accessible surface area
ΔASA: accessible solvent area
IFT: interface tag
NR: nonredundant
PDB: Protein Data Bank
PQS: Protein Quaternary Structure
RCC1: regulator of chromosome condensation
SCOP: Structural Classification of Proteins
