# Supplementary material for: The Many Faces of Protein–Protein Interactions: A Compendium of Interface Geometry
Source: PLoS Comput Biol. 2006 Sep 29;2(9):e124. doi: 10.1371/journal.pcbi.0020124 (PMC1584320; doi:10.1371/journal.pcbi.0020124)
Supplement: Figure S1 — The number of faces for a 1 domain protein is calculated from the interface classification. The other distributions for 2, 3, 4 domain proteins are derived from that of 1 domain protein. For single domain proteins, we observe that more than 30% of the domain families display only one face, whereas the protein kinase catalytic subunit family (d.144.1.7) shows the most face diversity of 51 face types. As the number of domains in a protein increases, the distribution shifts towards a higher face average, since its domain faces add up. The average number of faces for a 1, 2, 3, 4 domain protein is 3.2, 6.4, 9.6, 12.8, respectively. (30 KB PDF) [file pcbi.0020124.sg001.pdf]

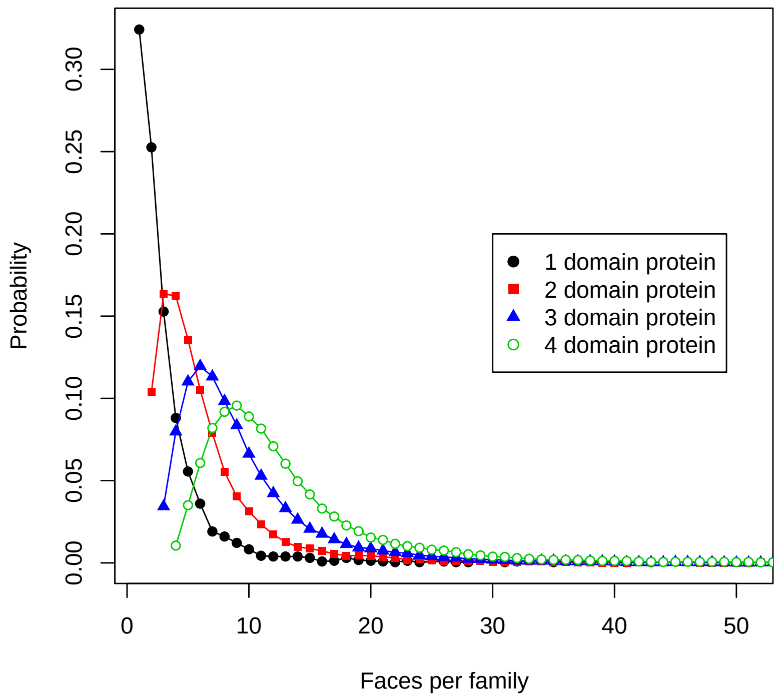

Figure S1. The probability distribution of the number of distinct face types per protein. The number of faces for a *1 domain protein* is calculated from the interface classification. The other distributions for 2, 3, 4 domain proteins are derived from that of *1 domain protein.* For single domain proteins, we observe that more than 30% of the domain families display only one face, whereas the protein kinase catalytic subunit family (d.144.1.7) shows the most face diversity of 51 face types. As the number of domains in a protein increases, the distribution shifts towards a higher face average, since its domain faces add up. The average number of faces for a 1, 2, 3, 4 domain protein is 3.2, 6.4, 9.6, 12.8, respectively.
