# Supplementary material for: The Many Faces of Protein–Protein Interactions: A Compendium of Interface Geometry
Source: PLoS Comput Biol. 2006 Sep 29;2(9):e124. doi: 10.1371/journal.pcbi.0020124 (PMC1584320; doi:10.1371/journal.pcbi.0020124)
Supplement: Figure S2 — (29 KB PDF) [file pcbi.0020124.sg002.pdf]

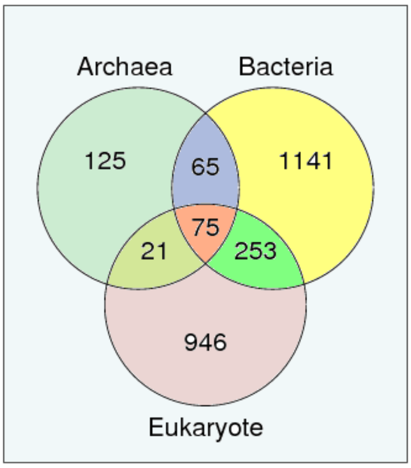


**Figure S2.** The taxonomic distribution of family pairs in PQS across the three kingdoms of life—Archea, Bacteria and Eukaryotes.
